# Elevation modulates the effects of tourism disturbance on soil fungal and bacterial communities in a cold-temperate montane forest

**DOI:** 10.3389/fmicb.2026.1915088

**Published:** 2026-09-07

**Authors:** Yifeng Cheng, Qun Lu, Xu Wang, Runchen Gao, Weiwei Xu, Xiaomin Fan, Zengyan Li, Xiaodong Yang

**Affiliations:** 1 School of Tourism, Xinjiang University of Finance and Economics, Ürümqi, China; 2 School of Geography and Remote Sensing, Ningbo University, Ningbo, China; 3 College of Ecology and Environment, Xinjiang University, Ürümqi, China

**Keywords:** elevational gradient, Kanas, soil microbial communities, soil physicochemical properties, tourism disturbance

## Abstract

Tourism activities in mountain forests can alter belowground ecological processes, yet how soil microbial communities respond to the combined effects of elevation and tourism disturbance remains unclear. Here, we investigated the diversity and composition of soil fungal and bacterial communities across different tourism disturbance intensities along an elevational gradient in the Kanas Lake forest area. Our results showed that both fungal and bacterial richness increased with elevation, while the effects of tourism disturbance on richness were modulated by elevation, with the lowest richness observed under high disturbance at the lowest elevation sites. Fungal richness was primarily associated with soil organic carbon (SOC), whereas bacterial richness was mainly related to soil water content (SWC) and total nitrogen (TN). Elevation significantly affected both fungal and bacterial community composition, whereas the interaction between elevation and tourism disturbance affected only fungal community composition. Compositional variation in both groups was related to SWC and SOC, with stronger correlations for fungi. Significant responses among dominant fungal taxa were detected only at the genus level, with *Mortierella* declining with increasing elevation and *Solicoccozyma* peaking at the middle elevation. Among dominant bacterial taxa, *Pseudomonadota* and *Actinomycetota* exhibited non-linear disturbance responses, with their highest mean relative abundances occurring under moderate disturbance, whereas *Chloroflexota* varied mainly with elevation. These findings suggest that areas at lower elevations and close to tourist trails are particularly sensitive to tourism disturbance, highlighting the need to reduce trampling and maintain soil habitat stability to support sustainable tourism management in mountain forest ecosystems.

## 1 Introduction

Mountain forests cover approximately 12% of the global land surface and play critical roles in carbon storage, water conservation, and the provision of raw materials, while also serving as important refugia for global biodiversity (Körner, 2007; Hagedorn et al., 2019). Mountain ecosystems are characterized by pronounced elevational gradients, where steep changes in temperature, precipitation, vegetation, and soil conditions can occur over relatively short geographic distances. They are therefore regarded as ideal natural laboratories for examining the effects of environmental gradients on biological communities (Körner, 2007; Teittinen et al., 2017). In recent decades, the rapid expansion of mountain tourism has introduced widespread anthropogenic disturbances, including trail construction, tourist trampling, and waste deposition, imposing persistent pressures on fragile mountain ecosystems (Pan et al., 2018; Niu and Cheng, 2019). Although previous studies have separately examined the effects of elevational gradients or tourism disturbance on biological communities (Praeg et al., 2020; Wang et al., 2020; Li et al., 2022), tourism activities in mountain scenic areas are often concentrated within specific elevational zones and along tourist trails, resulting in a close spatial coupling between elevation and tourism disturbance intensity. Elevation may therefore act not only as an environmental gradient but also as the ecological context in which tourism disturbance occurs. However, much less is known about whether elevational context modifies the magnitude and direction of belowground community responses to tourism disturbance.

Soil microorganisms constitute an important component of terrestrial biodiversity and play key roles in maintaining soil ecological functions and regulating ecosystem stability (Delgado-Baquerizo et al., 2016a; Delgado-Baquerizo et al., 2019). Owing to their short life cycles, active metabolism, and high sensitivity to environmental changes, soil microbial communities are widely regarded as sensitive indicators of environmental change in ecosystems (Guerra et al., 2021). Soil microbial diversity and community composition commonly vary along elevational gradients in relation to changes in soil moisture, organic carbon, pH, and nutrient availability (Tedersoo et al., 2014; Ochoa-Hueso et al., 2018; Praeg et al., 2019). In mountain tourism areas, distance from tourist trails is commonly used as a proxy for tourism disturbance intensity (Andrés-Abellán et al., 2005; Li et al., 2022). Areas closer to trails generally experience stronger and more frequent human disturbance, which can alter soil compaction, moisture, and nutrient availability, thereby affecting microbial diversity and community composition (Hu et al., 2011). Importantly, in resource-limited environments, such as soils with lower moisture and carbon availability, microorganisms are already under metabolic constraints, which may reduce their capacity to resist or recover from additional anthropogenic stress (Liao et al., 2023; Gao et al., 2025). As a result, the same intensity of tourism disturbance may produce stronger negative effects on microbial diversity at elevations with harsh soil conditions (Liao et al., 2023; Li et al., 2025).

Fungi and bacteria are major components of soil microbial communities (Zak et al., 2003; Strickland et al., 2009), yet they differ in resource acquisition strategies and sensitivities to changes in soil conditions (Yao et al., 2025). Bacterial nutrient acquisition depends strongly on water films surrounding soil particles, and their community composition often responds rapidly to variation in soil pH, moisture, and nutrient availability (Fierer and Jackson, 2006; Li et al., 2020). By contrast, many soil fungi form extensive hyphal networks and can utilize plant-derived and complex organic substrates, linking their community composition closely to the quantity and quality of soil organic matter (Drenovsky et al., 2004; Barnard et al., 2013). Because fungal communities tend to integrate environmental changes more persistently due to slower turnover, whereas bacterial communities may buffer disturbance effects through faster recovery (Barnard et al., 2013; Boyle et al., 2024), and because elevation and tourism disturbance can simultaneously modify soil physicochemical properties (Liao et al., 2023), the elevation-dependent effects of disturbance on community composition may differ between fungi and bacteria. In particular, the compositional responses of fungi to elevation and tourism disturbance may be more pronounced than those of bacteria. However, direct comparisons of fungal and bacterial community responses within the same elevation–disturbance framework remain limited.

Here, we examined how elevation and tourism disturbance jointly shape soil fungal and bacterial communities in typical mountain forests of the Kanas Lake area, Altai Mountains, Xinjiang. We established a blocked sampling design across three elevations, with replicate blocks at each elevation and plots located at varying distances from tourist trails to represent a gradient of disturbance intensity. Specifically, we aimed to determine: (a) how elevation and tourism disturbance independently and interactively affect fungal and bacterial *α*-diversity; and (b) how elevation and tourism disturbance reshape fungal and bacterial community composition and dominant taxa. We hypothesized that (a) tourism disturbance would have stronger negative effects on microbial diversity at elevations where baseline resource availability are lower, and (b) fungal community composition would show a stronger elevation-dependent response to tourism disturbance than bacterial communities, because fungi tend to accumulate environmental changes more persistently due to slower turnover, whereas bacteria may buffer disturbance effects through faster recovery. This study will provide a scientific basis for understanding the belowground ecological effects of tourism disturbance in mountain forests and for developing targeted management strategies that balance tourism development with ecological conservation.

## 2 Materials and methods

### 2.1 Study area

The study area is located in the Kanas Forest Area of the Altai Mountains, Xinjiang, China (86°54′–87°54′E, 48°35′–49°11′N). As a typical representative of China’s cold temperate coniferous and broadleaved mixed forest ecosystem, this forest area is regarded as the only region in the country that preserves a relatively intact southern taiga community of western Siberia (Cao et al., 2023). The zonal vegetation is dominated by taiga forests, with *Larix sibirica* and *Picea obovata* as the dominant tree species. The study area has a temperate continental monsoon climate, with a mean annual temperature of −0.2 °C. The mean temperature in the coldest month (January) is below −20 °C, and that in the warmest month (July) ranges from 15 to 18 °C. The annual precipitation is approximately 1,065 mm, showing the typical “wet island” climate characteristics in the arid region of Northwest China (Hou et al., 2024).

Since the initiation of tourism development in the 1980s, the Kanas Lake scenic area has gradually become a region with intensive tourism activities, receiving more than 3 million visitors in 2024. Tourism disturbance is spatially heterogeneous, generally decreasing from core scenic areas to peripheral areas and being concentrated along popular trails and scenic viewpoints. The coexistence of marked local elevational variation and tourism disturbance along trails makes this area suitable for exploring how natural environmental gradients and anthropogenic disturbance jointly shape soil microbial community structure.

### 2.2 Experimental design and sampling

Research plots were established along the tourist trail between Kanas Lake and the Fish Observation Platform. This trail section is located in the forest-mountain meadow ecotone of the Altai Mountains, where vegetation and landscape conditions change markedly within a relatively short elevational range. In addition, this section receives relatively high tourist visitation because of its distinctive landscape. Based on field accessibility, soil sampling feasibility, and the representativeness of the local vegetation transition, three elevational levels were selected at approximately 1800 m, 1900 m, and 2000 m, corresponding to low elevation (AL), middle elevation (AM), and high elevation (AH) within the investigated trail section, respectively. At each elevational level, three blocks were established along the same side of the tourist trail, with adjacent blocks separated by 20–30 m. Within each block, three 2 m × 2 m quadrats were arranged perpendicular to the trail edge, with their centers located 2, 5, and 20 m from the trail edge. These distances represented high (DH), moderate (DM), and low (DL) tourism disturbance intensities, respectively. Blocks were selected under similar slope aspect and vegetation conditions, yielding 27 plot-level sampling units in total (3 elevations × 3 blocks per elevation × 3 distances per block).

Soil sampling was conducted on a clear, rain-free day in September 2025. Before soil collection, surface litter and visible impurities were removed. In each quadrat, soil samples were collected from the 0–20 cm layer using a 5 cm diameter soil auger following the five point sampling method, and the five subsamples were thoroughly mixed to form one composite sample per quadrat. Each composite sample was divided into two subsamples: one was stored in a sterile sealed bag for the analysis of soil physicochemical properties, and the other was transferred into a sterile centrifuge tube and preserved at −80 °C for subsequent microbial DNA extraction and high throughput sequencing.

### 2.3 Soil physicochemical properties measurements

Soil physicochemical properties determined included SWC, pH, soil electrical conductivity (EC), bulk density (BD), SOC, total nitrogen (TN), and total phosphorus (TP). SWC was measured by the oven drying method. Soil pH and EC were determined using a pH meter and an electrical conductivity meter, respectively, at a soil:water mass ratio of 1:5. BD was measured using the core method, calculated as the mass of oven dried soil divided by the volume of the soil core. SOC was determined by the potassium dichromate external heating method, TN was measured using the Kjeldahl method, and TP was determined by the molybdenum antimony anti spectrophotometry. All measurement methods for soil physicochemical factors followed Cha (2017).

### 2.4 DNA extraction, PCR amplification, and Illumina sequencing

Soil microbial genomic DNA was extracted from soil samples stored at −80 °C using the PowerSoil^®^ DNA Isolation Kit according to the manufacturer’s instructions. DNA integrity was checked by 1% agarose gel electrophoresis to ensure no obvious degradation, and DNA concentration and purity were measured using a NanoDrop 2000 spectrophotometer. The V3–V4 region of the bacterial 16S rRNA gene was amplified using primers 338F (5’–ACTCCTACGGGAGGCAGCAG–3′) and 806R (5’–GGACTACHVGGGTWTCTAAT–3′). The fungal ITS1 region was amplified using primers ITS1F (5’–CTTGGTCATTTAGAGGAAGTAA–3′) and ITS2R (5’–GCTGCGTTCTTCATCGATG C–3′). The target regions were amplified by PCR using barcode-tagged primers to distinguish different samples. To minimize systematic errors caused by PCR amplification bias, the same optimized minimum cycle number, as determined by preliminary experiments, was used for all samples. PCR amplification was performed using TransStart FastPfu DNA polymerase (TransGen Biotech) on an ABI GeneAmp^®^ 9,700 thermal cycler. Each sample was amplified in triplicate. PCR products from the same sample were pooled and checked by 2% agarose gel electrophoresis, and target fragments were purified using the AxyPrep DNA Gel Extraction Kit. Purified PCR products were quantified using the QuantiFluor™-ST fluorometric system and mixed at an equimolar ratio to construct the sequencing library. Library preparation was performed with the Illumina TruSeq™ DNA Sample Prep Kit, including adapter ligation, quality control and denaturation. High-throughput sequencing was conducted on the Illumina platform using sequencing by synthesis (SBS) technology.

### 2.5 Bioinformatics analysis

Paired-end reads were quality-filtered using fastp v0.23.4 and merged using FLASH v1.2.11. The merged sequences were clustered into operational taxonomic units (OTUs) at 97% sequence identity similarity using UPARSE v11. Representative bacterial and fungal OTU sequences were taxonomically annotated against the SILVA v138 and UNITE v8 databases, respectively, using QIIME v1.9.1 with a confidence threshold of 0.7. Rarefaction curves based on observed OTU richness were generated to evaluate whether the sequencing depth was sufficient. To account for differences in sequencing depth among samples, the fungal and bacterial OTU tables were rarefied separately to 49,648 and 52,610 sequences per sample, respectively, based on the samples that yielded the lowest number of sequences for each kingdom after quality filtering. The resulting rarefied OTU tables were used for subsequent *α*-diversity and community composition analyses.

### 2.6 Statistical analyses

All statistical analyses were performed in R version 4.2.3. Microbial α-diversity was quantified using Chao1, Shannon–Wiener, and Simpson indices based on bacterial and fungal OTU tables. Linear mixed-effects models were used to test the main and interactive effects of elevation and tourism disturbance on soil physicochemical properties, microbial α-diversity indices, and relative abundances of dominant taxa. Elevation, tourism disturbance, and their interaction were included as fixed effects, whereas block identity nested within elevation was included as a random intercept to account for spatial non-independence. Model assumptions were evaluated by inspecting Q–Q plots and residual-versus-fitted plots. *Post hoc* comparisons were performed using Tukey’s HSD adjustment via the *emmeans* package.

Non-metric multidimensional scaling (NMDS) based on Bray–Curtis dissimilarities was used to visualize differences in fungal and bacterial community composition among elevation and tourism disturbance treatments. The main and interactive effects of elevation and tourism disturbance on community composition were tested using permutational multivariate analysis of variance (PERMANOVA) with 9,999 permutations. Block identity was included in the model to account for variation among spatial blocks. To account for the blocked sampling design, elevation was tested by permuting whole blocks, whereas permutations for tourism disturbance and the elevation × tourism disturbance interaction were restricted within blocks. NMDS and PERMANOVA analyses were conducted using the R package *vegan*.

Multiple linear regression was used to identify soil variables associated with *α*-diversity. All continuous variables used in the regression analyses were standardized using *z*-score transformation. For each α-diversity index, a full model including all candidate soil variables was constructed, and multicollinearity was checked using VIFs calculated with the *vif* function in the R package *car*. Since all VIF values were below 5, no candidate predictor was excluded because of multicollinearity. AIC-based stepwise selection was then performed from the full model using the *step* function in the R package *stats*, and the final AIC-selected model was used for interpretation. Mantel tests were performed to evaluate relationships between microbial community structure and soil physicochemical variables, with significance assessed using 999 permutations. Pearson correlation analysis was used to examine relationships among soil physicochemical properties, and between dominant microbial taxa and soil physicochemical properties.

## 3 Results

### 3.1 Soil physicochemical properties

Except for soil pH, elevation, tourism disturbance, and their interaction significantly affected all other soil physicochemical properties (*p* < 0.01, Table 1). SWC and SOC were higher at middle and high elevations than at low elevation, but their responses to tourism disturbance were elevation-dependent. At lower elevations, tourism disturbance significantly reduced SWC and SOC, whereas at higher elevations, differences among disturbance levels were relatively small. BD decreased with increasing elevation, and this decreasing pattern was more pronounced under high tourism disturbance. EC, TN, and TP showed complex elevation and tourism disturbance interactions, with their higher values mainly occurring under moderate tourism disturbance; EC and TP peaked at middle elevation, whereas TN was relatively higher at middle and high elevations.

**Table 1:** Soil physicochemical properties under different elevations and tourism disturbance combinations in Kanas Lake forest area.

Treatment	SWC (%)	pH	EC (mS/cm)	BD	SOC (g/kg)	TN (g/kg)	TP (g/kg)
AL-DH	9.30 ± 0.20Bb	6.90 ± 0.20Aa	0.12 ± 0.02Ba	0.75 ± 0.01Aa	70.42 ± 1.37Cb	1.85 ± 0.04Bb	0.21 ± 0.01Bc
AL-DM	8.30 ± 0.40Cb	7.10 ± 0.10Aa	0.11 ± 0.01Ca	0.74 ± 0.00Aa	90.04 ± 5.61Ca	1.48 ± 0.24Cc	1.32 ± 0.09Ba
AL-DL	13.40 ± 1.00Aa	7.10 ± 0.10Aa	0.11 ± 0.02Ba	0.73 ± 0.00Aa	84.07 ± 3.89Ba	2.64 ± 0.15Aa	0.91 ± 0.12Bb
AM-DH	14.90 ± 0.5Aa	7.20 ± 0.10Aa	0.17 ± 0.02Bb	0.56 ± 0.06Bb	102.60 ± 8.65Bb	1.58 ± 0.09Cb	0.71 ± 0.00Ab
AM-DM	14.50 ± 0.20Aa	7.10 ± 0.40Aa	0.66 ± 0.04Aa	0.68 ± 0.00Aa	122.73 ± 1.60Ba	2.77 ± 0.24Ba	3.10 ± 0.34Aa
AM-DL	14.00 ± 1.50Aa	7.40 ± 0.10Aa	0.18 ± 0.03Ab	0.64 ± 0.03Ba	133.94 ± 6.47Aa	1.47 ± 0.10Cb	0.81 ± 0.09Bb
AH-DH	13.70 ± 1.1Aa	7.10 ± 0.20Aa	0.24 ± 0.04Aa	0.46 ± 0.00Cb	126.18 ± 5.69Aa	2.60 ± 0.21Ab	0.96 ± 0.07Ab
AH-DM	10.40 ± 1.50Bb	7.10 ± 0.30Aa	0.23 ± 0.02Ba	0.70 ± 0.07Aa	135.73 ± 0.70Aa	4.01 ± 0.16Aa	1.31 ± 0.12Ba
AH-DL	15.20 ± 0.90Aa	7.10 ± 0.10Aa	0.16 ± 0.04ABb	0.49 ± 0.01Cb	124.91 ± 10.19Aa	2.12 ± 0.03Bc	1.43 ± 0.06Aa
Elevation	*p* < 0.001	*p* = 0.387	*p* < 0.001	*p* < 0.001	*p* < 0.001	*p* < 0.001	*p* < 0.001
Disturbance	*p* < 0.001	*p* = 0.290	*p* < 0.001	*p* < 0.001	*p* < 0.001	*p* < 0.001	*p* < 0.001
Elevation × Disturbance	*p* < 0.001	*p* = 0.295	*p* < 0.001	*p* < 0.001	*p* = 0.004	*p* < 0.001	*p* < 0.001

AL, AM, and AH represent low, middle, and high elevation, respectively; DH, DM, and DL represent high, moderate, and low tourism disturbance, respectively. Each combined label represents a specific elevation–disturbance treatment combination. SWC, soil water content; pH, soil pH value; EC, electrical conductivity; BD, bulk density; SOC, soil organic carbon; TN, total nitrogen; TP, total phosphorus. Different uppercase letters indicate significant differences among different elevations at same tourism disturbance intensity (*p* < 0.05), whereas different lowercase letters indicate significant differences among different tourism disturbance intensities at same elevation (*p* < 0.05).

### 3.2 Fungal and bacterial α-diversity

**Figure 1:**
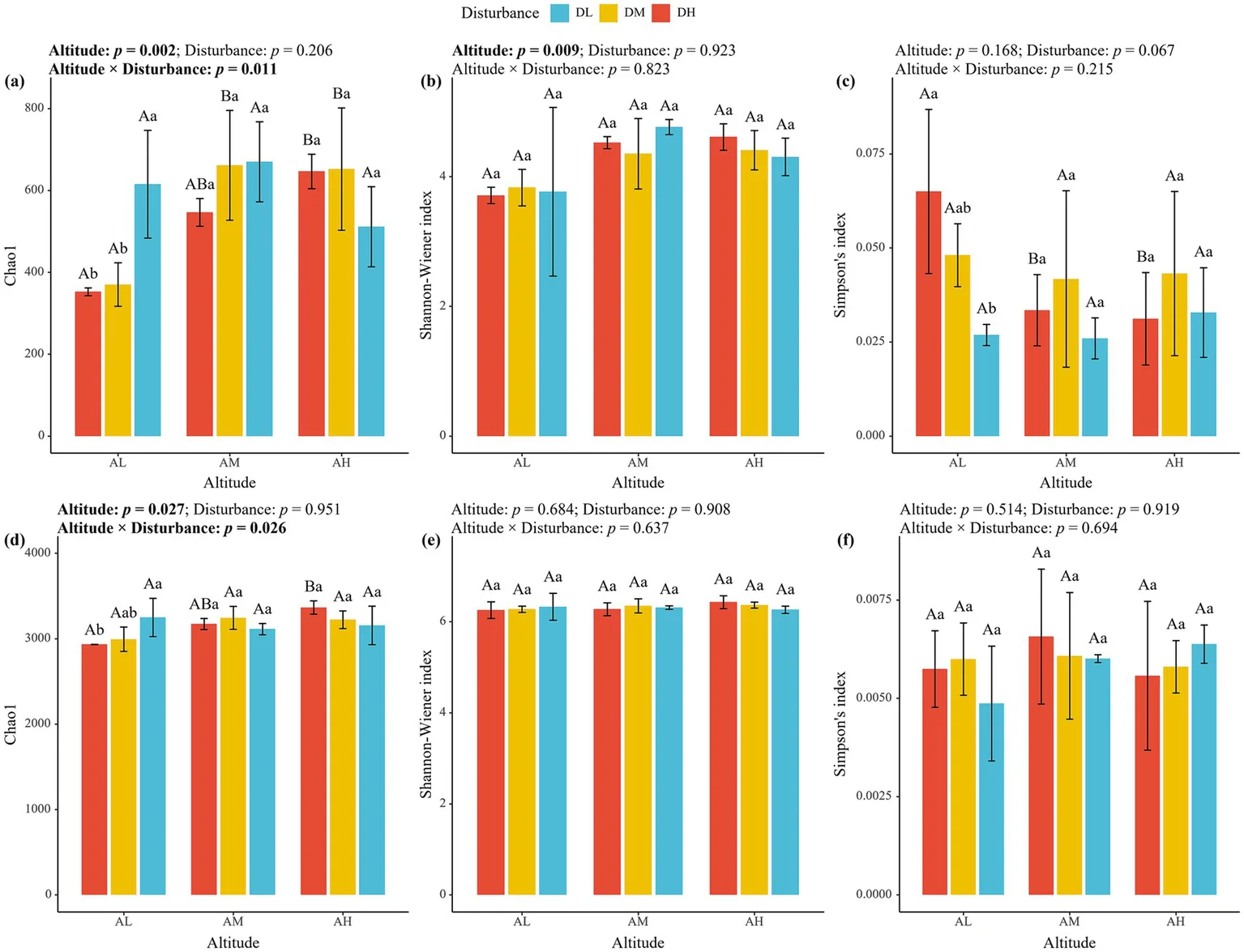
Fungal **(a–c)** and bacterial **(d–f)** *α* diversity indices along different elevational gradient and tourism disturbance intensity. Different uppercase letters indicate significant differences among different elevations at same tourism disturbance intensity (*p* < 0.05), whereas different lowercase letters indicate significant differences among different tourism disturbance intensities at same elevation (*p* < 0.05).

Rarefaction curves based on observed OTUs reached clear plateaus for all samples at the chosen rarefaction depths (Supplementary Figure S1), indicating that the sequencing depth was sufficient to capture the majority of microbial diversity. Across the 27 samples, fungal sequencing yielded a total of 3,869 operational taxonomic units (OTUs), with 211 to 763 OTUs detected per sample. Elevation and the interaction between elevation and tourism disturbance significantly affected the fungal Chao1 index (Figure 1a, *p* < 0.05). Specifically, under low disturbance, the Chao1 index did not differ significantly among elevation levels; however, under moderate and high tourism disturbance, it increased significantly with increasing elevation. At low elevation, tourism disturbance significantly decreased the fungal Chao1 index. Elevation also had a significant effect on the fungal Shannon–Wiener index (Figure 1b, *p* = 0.009), with higher values at the middle and high elevations than at the low elevation. No significant effects of elevation, tourism disturbance, or their interaction on the fungal Simpson index were detected (Figure 1c, *p* > 0.05).

For bacteria, a total of 6,127 operational taxonomic units (OTUs) were obtained, with 2,071 to 3,015 OTUs detected per sample. Elevation and the interaction between elevation and tourism disturbance significantly affected the bacterial Chao1 index (Figure 1d, *p* < 0.05). Specifically, the Chao1 index increased significantly with elevation under high disturbance, whereas it decreased significantly with increasing tourism disturbance at low elevation. Elevation, tourism disturbance, and their interaction had no significant effects on the Shannon–Wiener or Simpson indices of bacterial communities (Figures 1e,f; *p* > 0.05).

The best-fit model explained 41% of the variation in fungal Chao1 richness, which increased with SOC (*β* = 0.40, *p* = 0.043). Fungal Shannon–Wiener diversity was positively associated with SOC (*β* = 0.56, *p* = 0.002), and the corresponding model explained 29% of the variation. The fungal Simpson index decreased with increasing SWC (*β* = −0.41, *p* = 0.044), with the final model explaining 26% of the variation. For bacteria, the best-fit model explained 38% of the variation in Chao1 richness, which increased with both SWC and TN (SWC: *β* = 0.48, *p* = 0.005; TN: *β* = 0.46, *p* = 0.006). In contrast, soil physicochemical variables did not significantly explain variation in bacterial Shannon–Wiener or Simpson diversity (both *p* > 0.05; Table 2).

**Table 2:** Best-fit multiple regression models explaining the effects of soil physicochemical factors on fungal and bacterial α-diversity.

Taxa	Variable	Estimate	Std. error	*t*-value	*p*
Fungal α diversity	Chao1	*R*^2^_adj_ = 0.41, *p* = 0.002			
	SWC	0.25	0.17	1.46	0.157
	SOC	0.40	0.19	2.15	0.043
	TN	0.28	0.17	1.68	0.106
	Shannon–Wiener Index	*R*^2^_adj_ = 0.29, *p* = 0.002			
	SOC	0.56	0.17	3.40	0.002
	Simpson index	*R*^2^_adj_ = 0.26, *p* = 0.020			
	SWC	−0.41	0.19	−2.14	0.044
	EC	0.32	0.19	1.72	0.099
	SOC	−0.35	0.20	−1.76	0.093
Bacterial α diversity	Chao1	*R*^2^_adj_ = 0.38, *p* = 0.001			
	SWC	0.48	0.15	3.13	0.005
	TN	0.46	0.15	3.01	0.006
	Shannon–Wiener index	*R*^2^_adj_ = 0.04, *p* = 0.231			
	pH	−0.31	0.20	−1.50	0.146
	SOC	0.29	0.20	1.39	0.176
	Simpson index	*R*^2^_adj_ = 0.10, *p* = 0.116			
	pH	0.31	0.19	1.63	0.116

### 3.3 Fungal and bacterial community composition

**Figure 2:**
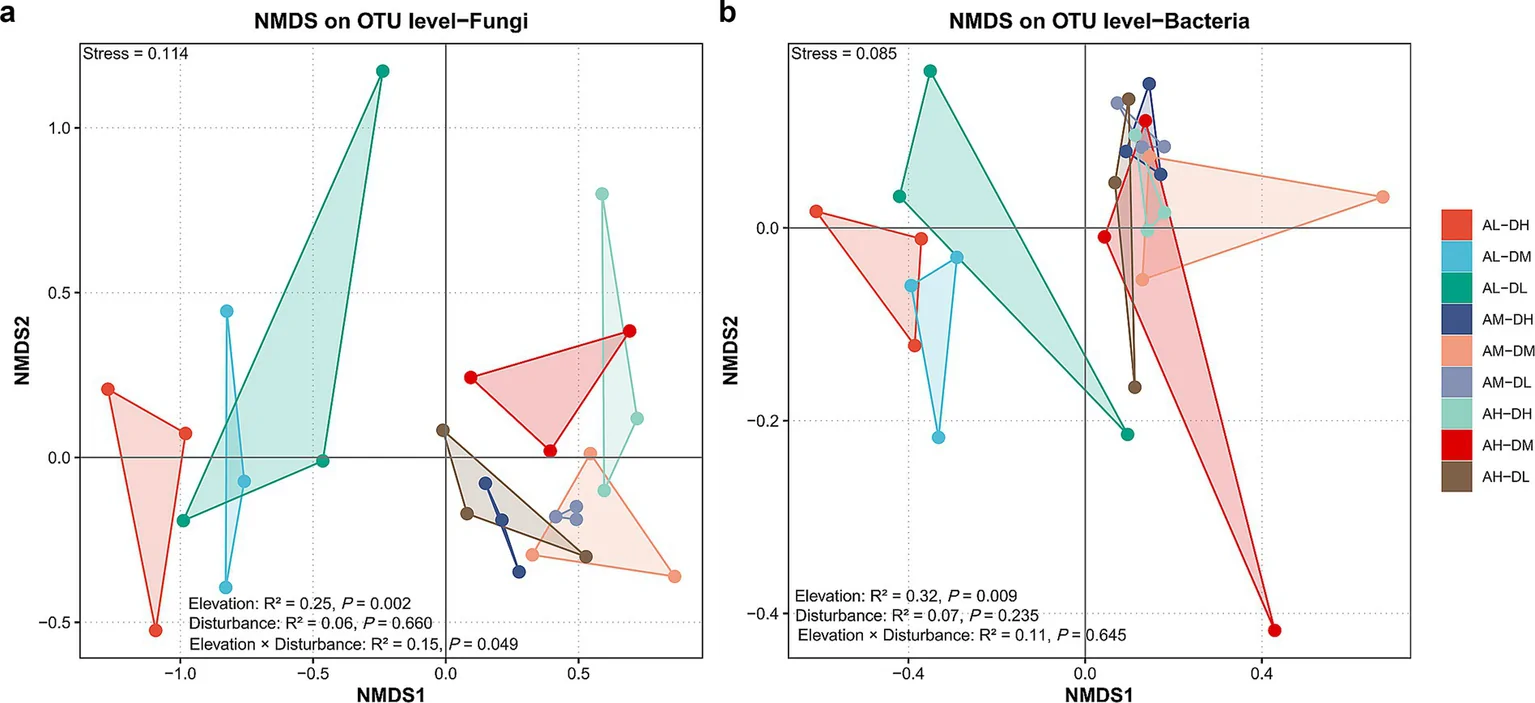
Two-dimensional non-metric multidimensional scaling (NMDS) ordination of soil fungal **(a)** and bacterial **(b)** communities.

In the ordination space, low-elevation fungal and bacterial communities were generally separated from those at the middle and high elevations, whereas the latter two showed considerable overlap (Figure 2). PERMANOVA confirmed significant differences in both fungal and bacterial community composition among elevations. Elevation explained 25% of the variation in fungal community composition (*p* = 0.002) and 32% of the variation in bacterial community composition (*p* = 0.009). In contrast, tourism disturbance had no significant main effect on either fungal or bacterial community composition (fungi: R^2^ = 0.06, *p* = 0.660; bacteria: *R*^2^ = 0.07, *p* = 0.235). For fungi, the relative positions and degree of overlap among disturbance treatments differed across elevations, consistent with a significant interaction between elevation and tourism disturbance (*R*^2^ = 0.15, *p* = 0.049). This result indicates that fungal community responses to tourism disturbance varied among elevations. No significant interaction was detected for bacterial communities (*R*^2^ = 0.11, *p* = 0.645).

**Figure 3:**
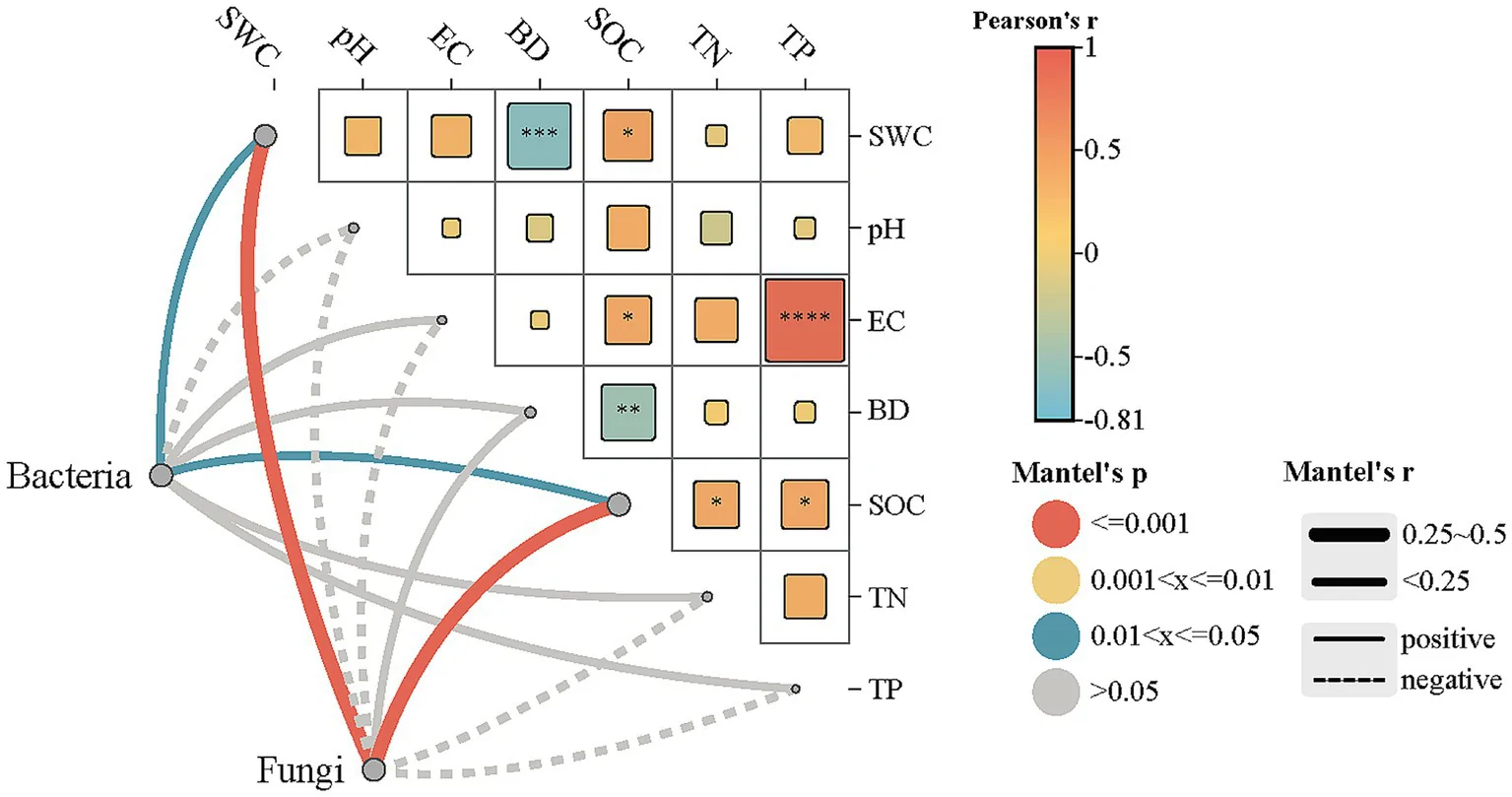
Correlation analysis of different soil physicochemical properties with the structure of soil microbial communities. *, **, *** and **** indicate significance level at 0.05, 0.01, 0.001 and 0.0001 respectively.

Pearson correlation analysis showed that BD was negatively correlated with SWC (Figure 3; *p* < 0.001), whereas SOC was positively correlated with SWC (*p* < 0.05). EC was positively correlated with both TP (*p* < 0.0001) and SOC (*p* < 0.05). In addition, BD was negatively correlated with SOC (*p* < 0.01), while SOC was positively correlated with TP and TN (*p* < 0.05). Mantel tests revealed that the composition of both fungal and bacterial communities was positively correlated with SWC and SOC (*p* < 0.05), with stronger correlations observed for fungal communities (0.25 < Mantel’s *r* < 0.50) than for bacterial communities (Mantel’s *r* < 0.25).

### 3.4 Relative abundances of dominant fungal and bacterial taxa

**Figure 4:**
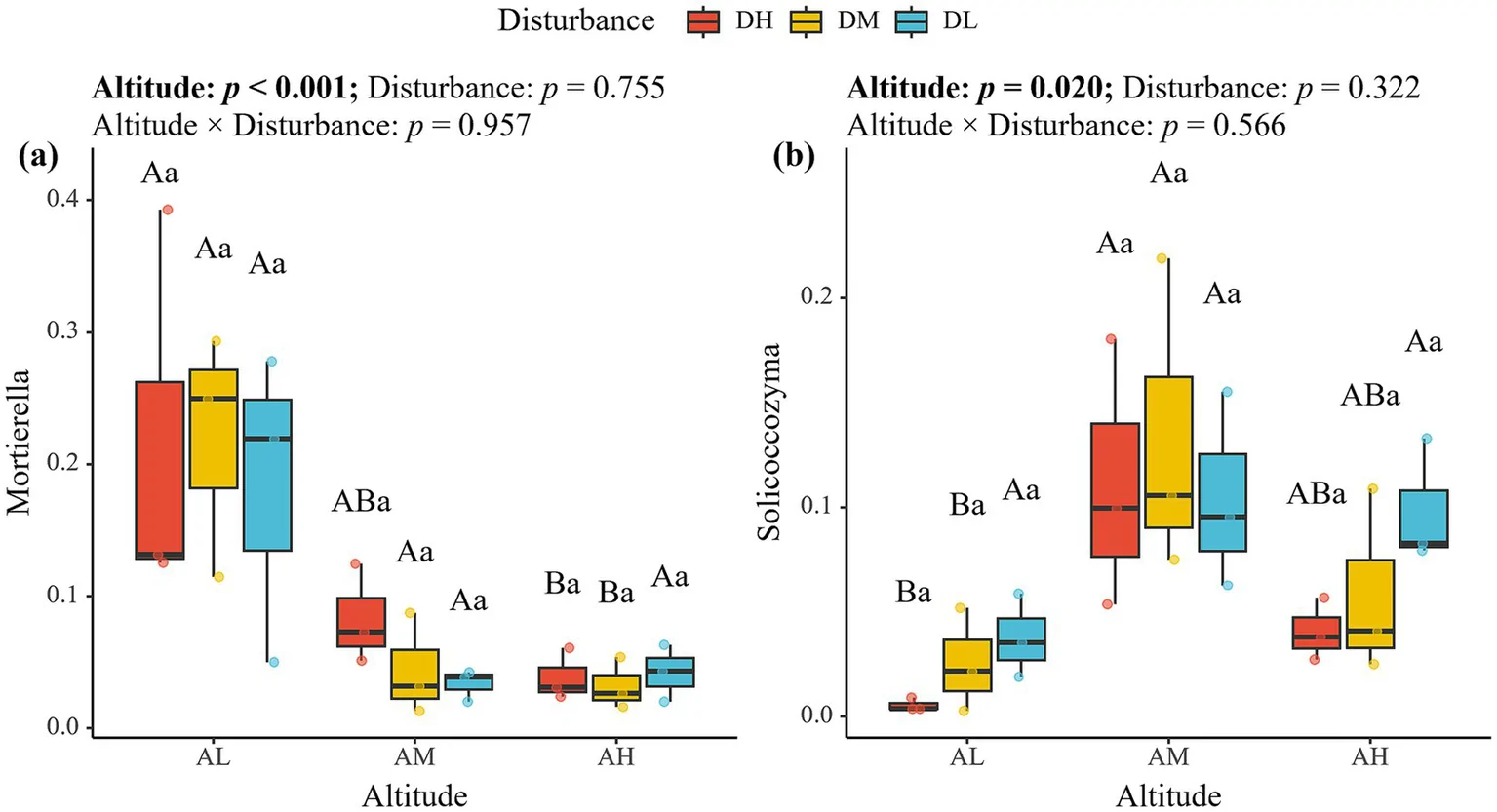
Relative abundances of dominant fungal taxa along different elevational gradient and tourism disturbance intensity. Different uppercase letters indicate significant differences among different elevations at same tourism disturbance intensity (*p* < 0.05), whereas different lowercase letters indicate significant differences among different tourism disturbance intensities at same elevation (*p* < 0.05).

Within the kingdom fungi (18 phyla), the community was dominated by *Ascomycota*, *Basidiomycota*, and *Mortierellomycota*, whose relative abundance were 52.85, 19.31, and 17.95%, respectively (Supplementary Figure S2). The relative abundances of these dominant fungal phyla were not significantly affected by elevation, tourism disturbance, or their interaction (Supplementary Table S1). At the genus level, two dominant fungal genera were identified. *Mortierella* had an average relative abundance of 9.91%, and its relative abundance was significantly higher at low elevation than at middle and high elevations (Figure 4a). *Solicoccozyma* had an average relative abundance of 6.82%, and its relative abundance was significantly affected by elevation (Figure 4b). Its relative abundance was significantly higher at middle elevation (11.62%) than at low elevation (2.28%).

**Figure 5:**
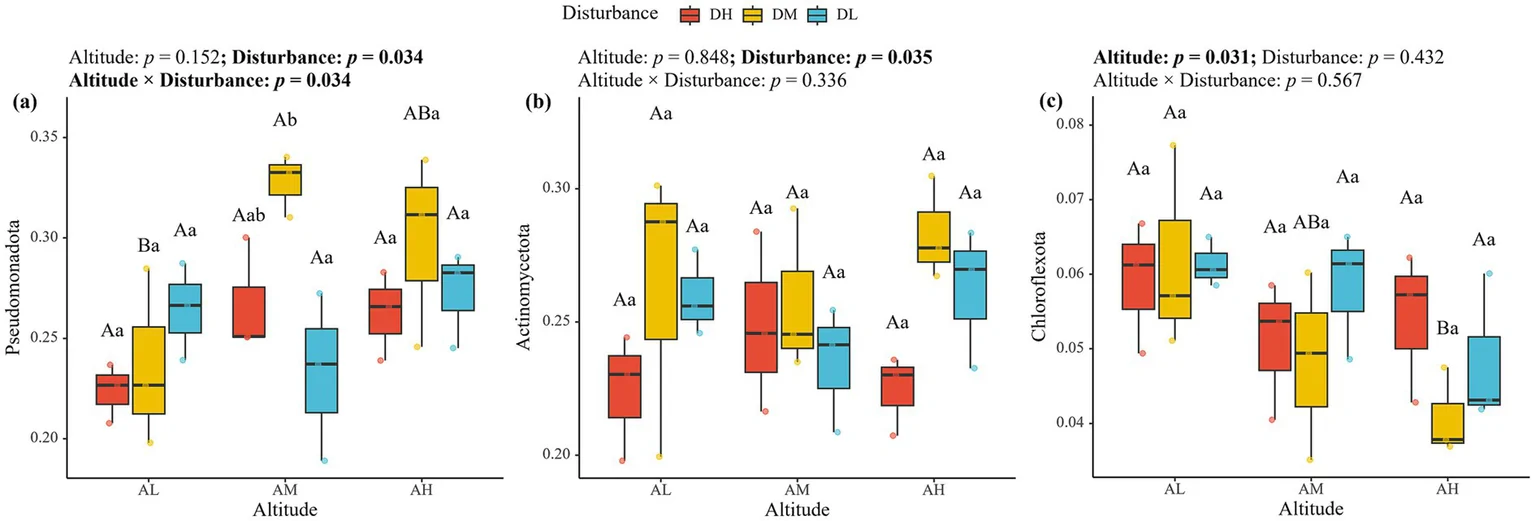
Relative abundances of dominant bacterial taxa along different elevational gradient and tourism disturbance intensity. Different uppercase letters indicate significant differences among different elevations at same tourism disturbance intensity (*p* < 0.05), whereas different lowercase letters indicate significant differences among different tourism disturbance intensities at same elevation (*p* < 0.05).

For bacteria, 36 phyla were detected. *Pseudomonadota* (26.51%), *Actinomycetota* (25.08%), *Acidobacteriota* (14.52%), *Verrucomicrobiota* (7.35%), *Bacteroidota* (6.90%), and *Chloroflexota* (5.37%) were the dominant phyla, together accounting for 84.79% of the bacterial community (Supplementary Figure S3a). At the genus level, the dominant taxa included *Candidatus Udaeobacter*, *norank_o_Vicinamibacterales*, and *norank_f_67* (Supplementary Figure S3b). Among the dominant bacterial taxa, significant responses were detected only for *Pseudomonadota*, *Actinomycetota*, and *Chloroflexota*. Tourism disturbance significantly affected the relative abundance of *Pseudomonadota*, with the magnitude of this effect varying among elevations (Figure 5a). Specifically, its relative abundance was highest under moderate disturbance, with differences among disturbance levels being most pronounced at the middle elevation. *Actinomycetota* also differed significantly among disturbance levels, with the highest mean relative abundance under moderate disturbance (Figure 5b). *Chloroflexota* differed significantly among elevations but showed no significant association with tourism disturbance (Figure 5c). No significant effects were detected for the remaining dominant bacterial taxa (Supplementary Table S1).

Pearson correlation analysis identified several significant associations between dominant microbial taxa and soil physicochemical properties (Table 3). Among fungal taxa, *Ascomycota* and *Mortierella* were negatively correlated with SWC and positively correlated with BD, whereas *Basidiomycota* showed the opposite pattern. *Mortierella* was also strongly negatively correlated with SOC, while *Solicoccozyma* was positively correlated with SWC, EC, SOC, and TP. Among bacterial taxa, *Pseudomonadota* was positively correlated with EC, TN, and TP, whereas *Chloroflexota* was negatively correlated with SOC and TN. Additional significant taxon–environment correlations are presented in Table 3.

**Table 3:** Correlations between the relative abundances of dominant taxa and environmental factors.

Taxa	Dominant taxa	SWC	pH	EC	BD	SOC	TN	TP
Fungi	*Ascomycota*	−0.52**	–	–	0.42*	–	–	–
	*Basidiomycota*	0.48*	–	–	−0.45*	–	–	–
	*Mortierellomycota*	–	–	–	–	–	−0.40*	–
	*Mortierella*	−0.48*	–	–	0.48*	−0.73***	–	–
	*Solicoccozyma*	0.58**	–	0.48*	–	0.43*	–	0.48*
Bacteria	*Pseudomonadota*	–	–	0.60***	–	–	0.52**	0.65***
	*Actinomycetota*	–	–	–	–	–	–	–
	*Acidobacteriota*	–	–	–	–	–	–	−0.48*
	*Verrucomicrobiota*	–	–	–	–	–	−0.42*	–
	*Bacteroidota*	–	–	–	–	0.40*	0.58**	–
	*Chloroflexota*	–	–	–	–	−0.38*	−0.43*	–
	*Candidatus Udaeobacter*	–	–	–	–	−0.53**	–	–
	*norank_o__Vicinamibacterales*	–	0.38*	–	−0.40*	–	–	–
	*norank_f__67*	–	0.51**	–	–	–	–	–

Only statistically significant correlations are shown. Values represent Pearson correlation coefficients. *, ** and *** indicate significance level at 0.05, 0.01 and 0.001, respectively.

## 4 Discussion

### 4.1 Fungal and bacterial α-diversity along elevational and tourism disturbance gradients

Consistent with our first hypothesis, the effects of tourism disturbance on fungal and bacterial richness depended on elevation, with the strongest reductions and the lowest richness occurring under high disturbance at low elevation. Both fungal and bacterial richness increased with elevation, a pattern consistent with Bryant et al. (2008) and Lyngwi et al. (2013), but contrasting with many studies reporting decreasing or unchanged microbial richness along altitudinal gradients (Fierer et al., 2011; Delgado-Baquerizo et al., 2016b; Yao et al., 2025). These contrasting patterns suggest that elevational trends in soil microbial diversity are highly context-dependent and may be governed by different environmental controls across ecosystems (Delgado-Baquerizo et al., 2016b; Cheng et al., 2026). Tourism disturbance reduced both fungal and bacterial richness, with stronger negative effects at low elevations, indicating that its impacts on soil microbial richness were elevation-dependent. The suppressive effects of tourism disturbance on microbial richness may be primarily attributed to stronger soil compaction under high tourism disturbance (near trails), caused by trampling (Baldrian, 2017; Nelson et al., 2024), which in turn reduce soil moisture and nutrient availability (Table 1). This effect may be amplified at low elevations, where soil water and nutrient conditions were relatively less favorable, making microbial communities more sensitive to additional disturbance (Li et al., 2025). Consequently, the combination of high tourism disturbance and poorer soil habitat conditions at low elevation likely contributed to the lowest microbial richness observed in these sites.

However, fungal and bacterial richness showed contrasting responses to environmental factors associated with elevation and tourism disturbance. In this study, SOC concentrations were lowest in the high-disturbance plots at low elevation and were positively associated with fungal richness. Although SWC and TN also exhibited lower values at low elevation under high disturbance and were retained in the final model, neither showed a significant individual association with fungal richness. In contrast, SWC and TN have significant effects on bacterial richness, and these effects are stronger than those on fungal richness, consistent with previous studies (de Vries et al., 2018; Ochoa-Hueso et al., 2018; Li et al., 2020). These contrasting responses may reflect fundamental differences in physiology and ecological strategies between fungi and bacteria. Hyphal foraging and the production of a broad repertoire of extracellular enzymes allow many fungi to exploit spatially heterogeneous resources and structurally complex organic substrates (Drenovsky et al., 2004; de Boer et al., 2005; Barnard et al., 2013). This may explain why fungal richness was significantly associated with SOC but not with SWC or TN. By contrast, many bacteria not only rely more heavily on dissolved substrates and exhibit relatively rapid resource turnover (Rousk and Bååth, 2011), but also have lower C to N biomass stoichiometry compared to fungi (Wallenstein et al., 2006). Their resource acquisition may therefore be particularly sensitive to the continuity of soil water films and to nitrogen availability, which may partly explain the observed association between bacterial richness and TN (Waring et al., 2013). Reductions in soil moisture can thin and disrupt these water films, thereby restricting the diffusion and accessibility of soluble substrates and nutrients (Stark and Firestone, 1995). Overall, fungal richness was primarily associated with SOC, whereas bacterial richness was associated with SWC and TN.

Notably, compared with Chao1 richness, the Shannon–Wiener and Simpson indices of fungal communities showed relatively weak responses to elevation and tourism disturbance, although tourism disturbance significantly increased the Shannon–Wiener index at low elevation. Because Chao1 is more sensitive to rare taxa, whereas Shannon–Wiener and Simpson indices are more strongly influenced by abundant taxa and community evenness, this pattern suggests that tourism disturbance may have primarily altered rare fungal taxa rather than broadly restructuring dominant taxa (Liu et al., 2020). The loss or reduction of rare fungal taxa under environmental stress may further weaken the potential functional breadth and resilience of fungal communities (Cui et al., 2023). The Shannon–Wiener and Simpson indices of bacterial communities exhibited no significant differences across elevation and disturbance gradients, suggesting that bacterial community evenness and dominance structure were less sensitive to these environmental gradients. This difference between fungi and bacteria may be partly attributed to the shorter generation times, higher metabolic flexibility, and potentially greater functional redundancy of bacteria, which may allow bacterial communities to maintain relatively stable diversity patterns under environmental fluctuations (Boyle et al., 2024; Luo et al., 2026).

### 4.2 Fungal and bacterial community composition and dominant taxa along elevational and tourism disturbance gradients

Our results showed that elevation significantly affected both fungal and bacterial community composition, whereas fungal responses to tourism disturbance differed among elevations, a pattern not observed for bacteria, partially supporting our second hypothesis. Three possible mechanisms may explain the contrasting responses of fungal and bacterial communities to tourism disturbance across elevations. First, soil environmental variables appeared to be more closely associated with fungal community composition than with bacterial community composition in this study. Mantel tests showed that both fungal and bacterial community composition were significantly and positively correlated with SWC and SOC, but these correlations were stronger for fungi than for bacteria (Figure 3). This suggests that changes in SWC and SOC associated with elevation and tourism disturbance may have contributed to shifts in fungal community composition, but were not sufficient to drive detectable changes in bacterial community composition. Second, bacteria exhibit greater resistance and resilience than fungi (Boyle et al., 2024), allowing bacterial communities to recover more quickly from disturbance and thereby buffering the elevation-dependent effects of disturbance on bacterial composition. Third, soil pH did not differ significantly among elevations or tourism disturbance levels. Because soil pH is widely recognized as an important environmental filter of bacterial community composition (Rousk et al., 2010; Shen et al., 2020), the absence of marked pH variation may have reduced one major source of bacterial compositional turnover in this study. Overall, these results suggest that the contrasting responses of fungal and bacterial communities to tourism disturbance across elevations can be explained by fungal coupling to moisture and carbon, bacterial buffering capacity, and relatively stable pH.

Community composition analysis further revealed taxonomic-level differences in fungal responses to elevation and tourism disturbance. The fungal community was dominated by *Ascomycota*, *Basidiomycota*, and *Mortierellomycota*, a pattern commonly observed in forest ecosystems (Shen et al., 2020; Fu et al., 2023; Cheng et al., 2026). The relative abundances of the dominant fungal phyla did not differ significantly with elevation or tourism disturbance. Instead, significant variation was detected at the genus level, particularly for *Mortierella* and *Solicoccozyma*. The relative abundance of *Mortierella* decreased with elevation. As a typical r-strategist saprotrophic fungal genus, *Mortierella* is often involved in organic matter decomposition and nutrient turnover. Its enrichment in low-elevation plots, where SOC and SWC were lower and BD was higher, suggests that this genus may have a competitive advantage in relatively dry, C-limited, and compacted soils (Simon et al., 2017). Functionally, *Mortierella* also contributes to soil aggregation and carbon stabilization through mineral-associated organic carbon pathways (Zhang et al., 2026); thus, its greater abundance at low elevation implies that carbon and nutrient cycling in these resource-limited soils may be disproportionately mediated by this genus. In contrast, *Solicoccozyma*, a psychrophilic yeast taxon, is more likely to occupy a dominant niche in mid-to-high elevation soils with low temperatures through cold-tolerant metabolic strategies and high resource utilization efficiency (Cantoran et al., 2025). Its enrichment at higher elevations suggests that yeast-mediated organic matter utilization may be more active in cooler soils, potentially influencing soil organic matter composition and turnover in these habitats. Overall, the contrasting elevational patterns of *Mortierella* and *Solicoccozyma* may suggest differences in environmental preferences and potential resource partitioning along the elevational gradient.

Among the dominant bacterial taxa, significant responses to elevation, tourism disturbance, or their interaction were detected only for *Chloroflexota*, *Actinomycetota*, and *Pseudomonadota*. Firstly, *Chloroflexota* decreased with increasing elevation and was negatively correlated with SOC and TN. *Chloroflexota* are involved in complex organic matter decomposition and nutrient cycling (Eichorst et al., 2018). Their greater relative abundance in low-elevation, resource-limited soils is consistent with oligotrophic adaptations, where resource-conservative strategies may confer a competitive advantage under carbon- and nitrogen-limited conditions (Kumar et al., 2024). Additionally, under moderate disturbance, *Chloroflexota* abundance was significantly lower at high elevation than at low elevation, suggesting that moderate disturbance may amplify the elevational decline of this phylum. Secondly, *Actinomycetota* showed no significant elevational trend but differed significantly among disturbance levels, with its highest mean relative abundance occurring under moderate disturbance. Similarly, *Pseudomonadota* exhibited its highest relative abundance under moderate disturbance and also showed an elevation-dependent response to tourism disturbance. This pattern is likely underpinned by concurrent changes in soil chemical properties. Our results showed that EC, TN, and TP were highest under moderate disturbance, and *Pseudomonadota* was positively correlated with these variables, suggesting that moderate tourism disturbance may modify litter fragmentation and local nutrient availability without imposing the stronger constraints that may occur under high disturbance (Chamara et al., 2024). Both *Actinomycetota* and *Pseudomonadota* are key decomposers contributing to litter decomposition and nutrient cycling (Mukhopadhya et al., 2012; Liu et al., 2019); their co-occurring peaks under moderate disturbance imply that decomposition and nutrient turnover may be transiently enhanced at intermediate disturbance levels. These functional inferences, however, are based on taxonomic assignments and require experimental validation.

### 4.3 Limitations and future directions

Several limitations should be considered when interpreting these findings. Our sampling followed an elevational gradient along a single trail, with three replicate blocks established at each elevation. This design enabled comparisons of elevation-dependent patterns within the investigated trail section, but did not provide independent replication across trails. Additionally, data were collected during only one sampling period. Consequently, the detected patterns may partly reflect trail-specific conditions or unmeasured spatial variation along the trail, and their temporal consistency remains to be evaluated. Future studies incorporating multiple independent trails and repeated sampling across seasons or years would help evaluate the spatial and temporal generality of these findings.

## 5 Conclusion

This study demonstrated that elevation and tourism disturbance jointly shaped soil fungal and bacterial communities in the forest–mountain meadow ecotone of the Kanas Lake area. Microbial richness generally increased with elevation, whereas the effect of tourism disturbance was strongly elevation-dependent. The lowest fungal and bacterial richness occurred under high disturbance at low elevation, indicating that habitats close to trails at low elevation are particularly vulnerable to human disturbance. Beyond α-diversity, fungi and bacteria responded differently in community composition and dominant taxa. Fungal communities exhibited significant compositional shifts across elevation and tourism disturbance treatments, and the responses of dominant fungal taxa were mainly reflected in the opposite elevational trends of *Mortierella* and *Solicoccozyma*. By contrast, bacterial community composition responded mainly to elevation, with little evidence for an elevation-dependent disturbance effect, whereas dominant bacterial taxa showed pronounced nonlinear shifts in relative abundance, especially under moderate tourism disturbance. Soil physicochemical properties, particularly SWC, SOC, and TN, played important mediating roles in these patterns by linking elevation and tourism disturbance to microbial richness and community composition. Overall, these findings indicate that microbial associations with tourism disturbance depend on elevational context and differ between fungal and bacterial communities. From a management perspective, conservation efforts within the investigated trail section should prioritize lower-elevation, trail-adjacent areas, where resource-limited soil conditions may have heightened the negative effects of intensive tourism disturbance on fungal and bacterial richness.

## Data Availability

The original contributions presented in the study are publicly available. This data can be found in the NCBI Sequence Read Archive under the accession number PRJNA1520501.
