# Supplementary material for: Elevation modulates the effects of tourism disturbance on soil fungal and bacterial communities in a cold-temperate montane forest
Source: Front Microbiol. 2026 Sep 7;17:1915088. doi: 10.3389/fmicb.2026.1915088 (PMC13590770; doi:10.3389/fmicb.2026.1915088)
Supplement: Supplementary file 1 [file Table_1.DOCX]

Supplementary Material

**Figure S1:** The sequencing depth for fungus (a) and bacterium (b)

**Figure S2:** Fungal community composition at the phylum (**a**) and genus (**b**) levels.

**Figure S3:** Bacterial community composition at the phylum (**a**) and genus (**b**) levels.

**Table S1:** The effects of elevation, tourism disturbance, and their interaction on the relative abundances of dominant microbial taxa in the Kanas Lake forest area.

| Factors | *Ascomycota* | |  | *Basidiomycota* | |  | *Mortierellomycota* | |  | *Mortierella* | |  | *Solicoccozyma* | |
| --- | --- | --- | --- | --- | --- | --- | --- | --- | --- | --- | --- | --- | --- | --- |
|  | F-value | *p*-value |  | F-value | *p*-value |  | F-value | *p*-value |  | F-value | *p*-value |  | F-value | *p*-value |
| Elevation | 1.19 | 0.327 |  | 2.64 | 0.099 |  | 1.93 | 0.225 |  | 13.94 | <0.001 |  | 8.01 | 0.020 |
| Disturbance | 1.69 | 0.213 |  | 0.36 | 0.699 |  | 0.21 | 0.815 |  | 0.29 | 0.755 |  | 1.25 | 0.322 |
| Elevation × Disturbance | 1.66 | 0.203 |  | 1.02 | 0.422 |  | 0.66 | 0.630 |  | 0.16 | 0.957 |  | 0.77 | 0.566 |
| Factors | *Pseudomonadota* | |  | *Actinomycetota* | |  | *Acidobacteriota* | |  | *Verrucomicrobiota* | |  | *Bacteroidota* | |
|  | F-value | *p*-value |  | F-value | *p*-value |  | F-value | *p*-value |  | F-value | *p*-value |  | F-value | *p*-value |
| Elevation | 2.63 | 0.152 |  | 0.17 | 0.848 |  | 0.42 | 0.673 |  | 2.32 | 0.127 |  | 3.07 | 0.071 |
| Disturbance | 4.54 | 0.034 |  | 4.51 | 0.035 |  | 3.69 | 0.056 |  | 0.24 | 0.787 |  | 1.10 | 0.353 |
| Elevation × Disturbance | 3.76 | 0.033 |  | 1.27 | 0.336 |  | 0.21 | 0.926 |  | 1.20 | 0.343 |  | 1.29 | 0.309 |
| Factors | *Chloroflexota* | |  | *Candidatus Udaeobacter* | |  | *norank_o__Vicinamibacterales* | |  | *norank_f__67* | |  |  |  |
|  | F-value | *p*-value |  | F-value | *p*-value |  | F-value | *p*-value |  | F-value | *p*-value |  |  |  |
| Elevation | 4.22 | 0.031 |  | 4.37 | 0.067 |  | 2.22 | 0.190 |  | 0.08 | 0.922 |  |  |  |
| Disturbance | 0.88 | 0.432 |  | 0.15 | 0.861 |  | 2.59 | 0.116 |  | 1.50 | 0.250 |  |  |  |
| Elevation × Disturbance | 0.76 | 0.567 |  | 0.53 | 0.714 |  | 0.51 | 0.733 |  | 2.13 | 0.119 |  |  |  |
